# Factors Affecting Microalgae Production for Biofuels and the Potentials of Chemometric Methods in Assessing and Optimizing Productivity

**DOI:** 10.3390/cells8080851

**Published:** 2019-08-07

**Authors:** Mutah Musa, Godwin A. Ayoko, Andrew Ward, Christine Rösch, Richard J. Brown, Thomas J. Rainey

**Affiliations:** 1 Biofuel Engine Research Facility, School of Chemistry, Physics and Mechanical Engineering, Science and Engineering Faculty, Queensland University of Technology (QUT), Queensland 4000, Australia; 2 Environmental Technologies Discipline, School of Chemistry, Physics and Mechanical Engineering, Science and Engineering Faculty, Queensland University of Technology, Queensland 4000, Australia; 3 Queensland Urban Utilities (QUU), Innovation Centre, Main Beach Road Myrtletown QLD 4008, Australia; 4 Advanced Water Management Centre (AWMC), University of Queensland (UQ), St Lucia, Brisbane, Queensland 4072, Australia; 5 Institute for Technology Assessment and Systems Analysis (ITAS), Karlsruhe Institute of Technology, 76021 Karlsruhe, Germany

**Keywords:** microalgae, chemometrics, lipids, biofuels, biorefinery, multivariate analysis, pattern recognition, process optimization

## Abstract

Microalgae are swift replicating photosynthetic microorganisms with several applications for food, chemicals, medicine and fuel. Microalgae have been identified to be suitable for biofuels production, due to their high lipid contents. Microalgae-based biofuels have the potential to meet the increasing energy demands and reduce greenhouse gas (GHG) emissions. However, the present state of technology does not economically support sustainable large-scale production. The biofuel production process comprises the upstream and downstream processing phases, with several uncertainties involved. This review examines the various production and processing stages, and considers the use of chemometric methods in identifying and understanding relationships from measured study parameters via statistical methods, across microalgae production stages. This approach enables collection of relevant information for system performance assessment. The principal benefit of such analysis is the identification of the key contributing factors, useful for decision makers to improve system design, operation and process economics. Chemometrics proffers options for time saving in data analysis, as well as efficient process optimization, which could be relevant for the continuous growth of the microalgae industry.

## 1. Introduction

**Figure 1:**
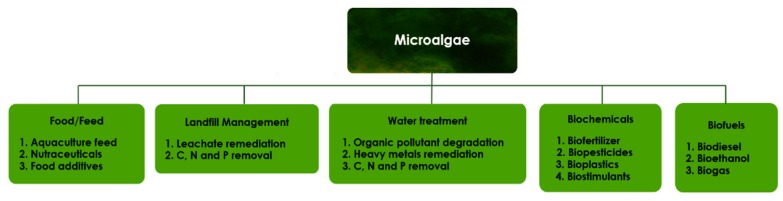
Uses and products of microalgae.

Microalgae are microorganisms that swiftly replicate through photosynthesis, absorbing light in the presence of nutrients and CO_2_. Microalgae has high CO_2_ sequestration efficiency and have the additional environmental benefit in its ability to utilize nutrients to remediate wastewater. Microalgae are also known to have high oil yields in comparison with other biofuel feedstock crops like palm oil and soybeans [1,2]. The potential to derive several co-products from microalgae also makes it a high value commodity (Figure 1).

In recent times, there has been increased interest on microalgae for low value products (e.g., biofuels), as well as high value products (e.g., nutraceuticals) both within research institutes and industry. The predicted depletion of fossil fuels and environmental concerns related to climate change is driving the quest for renewable, clean and sustainable sources of energy [3,4]. Microalgae suitably meets this criterion, however the dilute state in which it is cultivated and its small cell size creates several challenges against its economic production and processing to biofuels. Common problem areas in the production and processing of microalgae relate to strain selection and improvement, inefficient use of sunlight for photosynthesis in ponds, the need to remove large volumes of water to obtain concentrates, as well as conversion and processing into biofuels or other products listed in Figure 1.

Chemometrics refers to the technique that involves the use of optimal mathematical and statistical methods in order to extract and correlate chemical and physical information and how they affect a process or the outcome of the process [5]. Chemometrics allows several non-selective signals to be combined in a multivariate model to measure a desired information with high precision [6]. A common problem experienced in chemistry, engineering and the environmental sciences as a whole is that related to process optimization [7]. The traditional approach of one-factor-at-a-time (OFAT) experiments, where it is possible to systematically vary one factor while holding others constant in a process is commonly used to study the effect and contribution of individual factors in chemical and physical processes. Such an approach could be time consuming and does not take into account multifactor interactions [8]. On the other hand chemometrics could be deployed to analyze multidimensional interactions of a non-linear and non-selective form [6]. Chemometric data interpretation provides further and clearer information that cannot be derived through conventional statistical data interpretation for any given data set. It is noteworthy to state that it does not however replace best practice in the process of data collection and interpretative analysis [9]. The application of chemometrics in assessing and analyzing the production and processing microalgae will be considered in this review.

## 2. Renewable Energy and the Prospects of Microalgae Biofuels

**Figure 2:**
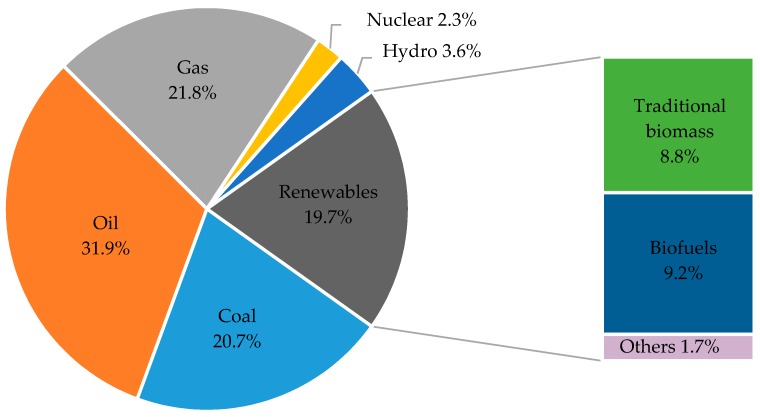
Total global primary energy demand by source (others includes wind, solar and geothermal energy) [11].

Renewable energy is the energy that comes from sources, which are continuously replenished by nature. Currently, renewable energy contributes far below its expected capacity to total global energy production. Renewable energy only meets ~20% of global energy demand as shown in Figure 2 [10,11]. This emerging sources of energy comprise wind, solar and hydro-, geothermal, biomass, waves and tidal energy [12]. Solar energy is an appealing source of energy because of its high magnitude and availability through radiant light and heat from the sun. If efficiently harnessed it has the potential of meeting global electricity demand, and it is increasingly being adopted for use world-wide. The National Renewable Energy Laboratory (NREL) has reported solar panel efficiencies exceeding 45%, which demonstrates the potential role of solar energy in the future of power generation [13]. Solar energy is mainly being exploited for electricity generation and water heating, with limited use in the transport sector.

Although there is increasing generation of electricity from solar and other sources of renewable energy, the transport sector is still heavily dependent on fossil fuels and will continue to use them well into the future. When comparing solar conversion efficiency, theoretically a photovoltaic installation can produce 20–30 times more power per square meter of land than biodiesel sourced from a hypothetical high yield switchgrass [14]. When considering vehicle transport, liquid fuels provide greater flexibility when compared to electric vehicles. Previous studies suggest that plug-in hybrids electric vehicles (PHEV), battery electric vehicles (BEV), fuel cell vehicles (FCV) and biofuels will all have a significant role to play in the attainment of policy goals focused on cutting down emissions from the transport sector [15,16]. Furthermore, previous indications demonstrated that PHEVs may be constrained by the availability of sustainable biofuels [16]. Therefore, there is a need to continuously match-up the development of the biofuels industry, through efficient energy saving and low cost processes.

**Figure 3:**
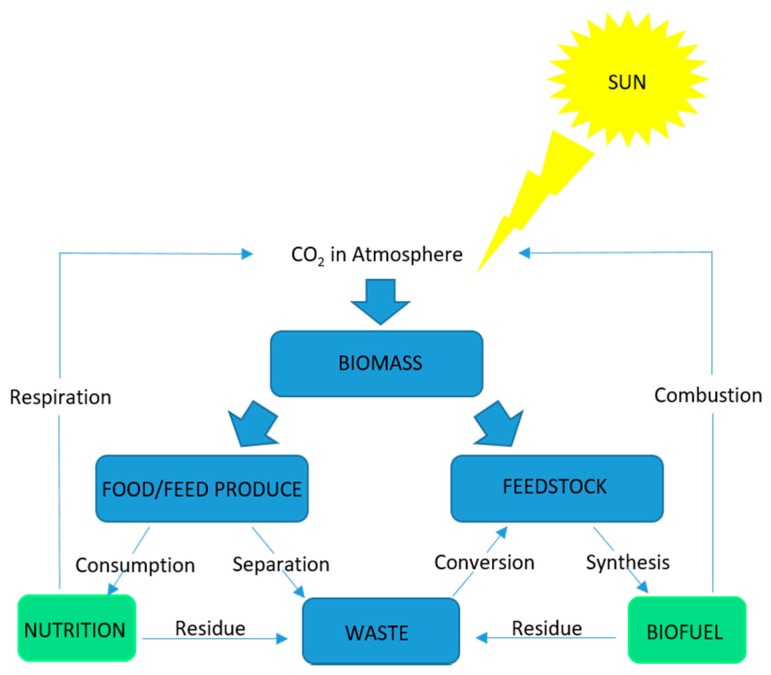
Main features of biomass energy technology.

In furtherance of the quest for renewable energy sources, there has been initiatives to move the global economy from being fossil-fuel based to a bio-based economy [17]. Carbon sources are considered the most suitable replacement for fossil fuels in the transport sector [18]. The only renewable carbon source large enough to replace fossil fuel is biomass. As biomass contains all the elements found in fossil fuels, albeit in different proportions, present and developing technologies in biomass production and processing can lead to sustainable, low carbon emission economies driven by renewable energy [19]. The key biomass production process involves the capture of solar energy to fix carbon through photosynthesis [20]. Chemical energy from the process is biologically stored as organic materials synthesized from CO_2_ and H_2_O, this reaction is represented by Equation (1). The pathways for the conversion of biomass into renewable energy sources are further illustrated in Figure 3. Multiple biomass feedstock sources have been explored and are still under investigation for biofuel production. Biomass produced biofuels have been categorized into generations based on their origin and exploration era.

(1) $${6\mathrm{CO}}_{2}{+6H}_{2}O+\mathrm{Light}\;\mathrm{energy}\overset{\mathrm{yields}}{\to }\;\underset{\mathrm{Organic}\;\mathrm{materials}}{C_{6}H_{12}O_{6}}{+O}_{2}.$$

The feedstock for the first generation biofuels are mainly sourced from food crops. The products (biodiesel and bioethanol) were mostly abstracted through fermentation and transesterification of sugars, starches and oils, which were readily available in food sourced biomass. Wheat, sugarcane, corn, soybeans and rapeseed were among the first generation food based feedstocks. The debatable concern about the use of food crops was whether they actually contributed in reducing greenhouse gas (GHG) emissions, considering their high production CO_2_ footprint. Another concern with the-use of food crop biomass was their diversion from the global food market, raising the fuel versus food debate.

Second generation biofuels were developed out of the need to address the concerns raised from the fuel versus food debate associated with first generation biofuels. Thus, the focus was on non-food crop and waste sourced biomass. Examples of feedstocks for this generation are switchgrass, jatropha, waste vegetable oils, municipal solid waste, sugarcane bagasse as well as a range of other lignocellulosic agricultural wastes. The second generation biofuel feedstocks are considered to have eliminated the problems associated with the first generation biofuels and are economically competitive with fossil fuels [21]. The attributed challenge however, is the wide diversity of composition, which is associated with varying processing requirements for feedstock of different origins. Therefore, it is objectively difficult to recommend an ideal feedstock for biofuels production due to variations in terms of technology, performance, operating conditions, scale, target markets, and variations found in pilot and commercial scale analysis available in the scientific literature. For instance, factors such as geographical location can have significant impact on production performance even for replicate systems using the same feedstock [22].

Algal biomass has been singled out as a feedstock of third generation biofuels. Yields reported are at least 10 fold higher than that obtainable from other lipid-rich agricultural crops [23]. Its biggest benefit is the ability to be modified and processed on a commercial scale to obtain different products in a biorefinery process approach. Algal biomass third generation of biofuels are divided into macro- and microalgae, and to some extent linked to the utilization of CO_2_ in the production of feedstock [24]. The production of liquid and gaseous biofuels from algae has been topical in the academia and industry, with the majority of research focused on microalgae [25]. Macroalgae also commonly known as seaweed has high applicability for biofuels to produce biodiesel, bioethanol, biobutanol and biogas. Earlier studies suggested that prioritizing biogas production was a more economically favorable approach to biofuels production from macroalgae [26]. Over the years, macroalgae have yielded high conversion efficiencies in the production of several other biofuels, including fermentative production of bioethanol using enzymatic hydrolysis, mostly due to higher carbohydrate contents [27], hydrothermal pretreatment followed by dark fermentation to produce biohydrogen and anaerobic digestion to produce biomethane [28]. Most algae species are unicellular and commonly referred to as microalgae. These group of algae are heterogeneous and have been used as food in ancient civilizations, with their taxonomic classification still ongoing [29]. Following the oil crisis in the early 1970s, several renewable energy programs were launched, including that for microalgae biofuels by the US Aquatic Species Program (i.e., ASP, currently NREL). The growth of the microalgae biofuels industry was promoted by the opportunities for GHG mitigation through CO_2_ capture using microalgae and the development of biofuel policies globally [29,30,31]. However, the key challenge to processing microalgae is the need for dewatering prior to conversion, which requires high amounts of energy and constitutes a significant processing cost, accounting for 20–30% of the total production cost [32]. These as well as other processing constraints have kept microalgae biofuels production technically possible but not economically feasible. In more recent times research has been reoriented towards the biorefinery concept for multiple products, thus increasing the economic feasibility of microalgae biofuels technology [29,33].

Due to microalgae’s high productivity per unit area of land and the ability to be cultivated in areas unsuitable for traditional agriculture, it is significantly more efficient when compared to most oil seed crops with reduced cultivation time [34]. Microalgae also has a potential to derive its nutrients from wastewater treatment facilities, and CO_2_ from flue gas discharge of coalmines or other combustion based power plants. Microalgae has received significant attention due to its facile adaptability for multiple applications, such as wastewater treatment and aquaculture [35]. Microalgae production is currently undergoing significant development both within research and industrial sectors as a third generation biofuel feedstock. This is due to its higher photosynthetic efficiency in biomass production, faster growth rate, non-competition with food crops for arable land use and high lipid yield, making a more sustainable feedstock when compared to first and second generation biofuels.

The microalgae biomass cultivation process produces very dilute suspension, therefore it needs to be dewatered prior to any further processing [22]. Dewatering is broadly divided into a primary stage of thickening and a secondary stage of dewatering to concentrate the biomass for further processing [36]. The end of use of the harvested material is important in the choice of harvesting technique. High purity is required for food and feed products. Subsequent processes like drying and cell disruption are important stages especially for lipid production. However, drying is considered uneconomic because of the high energy demand.

Several mechanical, chemical and enzymatic techniques have been developed for lipid extraction [31]. There is a wide variation in the literature about specific processes applied in lipid extraction, depending on technology dry or wet microalgae could be used in extraction with organic solvents. Solvents are often recycled after distillation and the extracted oil further processed. Biomass can also be processed through hydrothermal liquefaction (HTL) to produce biocrude i.e., renewable diesel blendstock (RDB) or processed via transesterification to obtain fatty acid methyl ester (FAME) biodiesel [30]. After the lipid extraction, residual biomass can also be further processed using anaerobic digestion to produce biogas with digestate solids and liquor from the process capable for use as biofertilizer [37] and microalgae cultivation recycled nutrients [30] respectively. When not anaerobically digested to obtain the products mentioned above, it is also possible to produce ethanol as another option using the residual biomass from the extraction process through hydrolysis and fermentation, but this is highly dependent on the carbohydrate content of the biomass [38].

## 3. Microalgae Productivity: Opportunities and Challenges

Photosynthesis is the primary energy production process for biomass sources of renewable energy. It is therefore based on this high photosynthetic productivity that the development of a microalgae biofuel industry could be built. Phototrophic cultivation, which is the photosynthetic conversion of CO_2_ with sunlight and nutrients in an open or closed system, is a key process to optimize in the economic production of microalgae. Heterotrophic production is also available but it comes with very high operational costs due to the requirement of a supplemented carbon source (e.g., sugar). Phototrophic production is perceived as the best route to attain sustainable biomass production. Microalgae can exist in two physiological types; low light adapted (with high chlorophyll content) and high light adapted (with low chlorophyll content). During photosynthesis light photons are absorbed by chlorophyll molecules to yield a charge separation that ejects electrons. These electrons in turn create water dissociation, generating protons and oxygen. This activity by the protons and electrons enables the reduction of CO_2_ to organic materials, which can be converted to protein, carbohydrates and lipids while oxygen is released as a by-product of the reaction [31]. The reaction that takes places is described by Equation (1). This process could be separated into two phases: A set of photochemical and redox reactions (light reactions) and a sequence of enzymatic reactions (light-independent reactions) presented as Equations (2) and (3). The light reactions operate on short timescales (milliseconds), while the light-independent reactions operate on longer timescales (seconds–hours). This disparity in timescales creates inefficiencies with fluctuations in temperature and irradiance, resulting in lower biomass yields [31]. In a study by Yarnold et al. [39], light illumination profile was found to significantly influence biomass productivity, with cycle times below 12 seconds modestly improving productivity. The study concluded that if maintained on very short timescales (femtoseconds–milliseconds), light and light-independent reactions will result in the ‘flashing light’ effect, which can improve photon conversion efficiency (PCE).

(2) $${\mathrm{Light}\;\mathrm{reaction}:\;2H}_{2}O\;\to 4\left[H\right]{+O}_{2}+\mathrm{energy},$$

(3) $$\text{Light-independent reaction}:\;4\left[H\right]\to {[C2H}_{2}{O]+H}_{2}O.$$

The notation [H] refers to the combination of the reduced coenzyme nicotinamide adenine dinucleotide phosphate (NADPH) and an electron).

**Figure 4:**
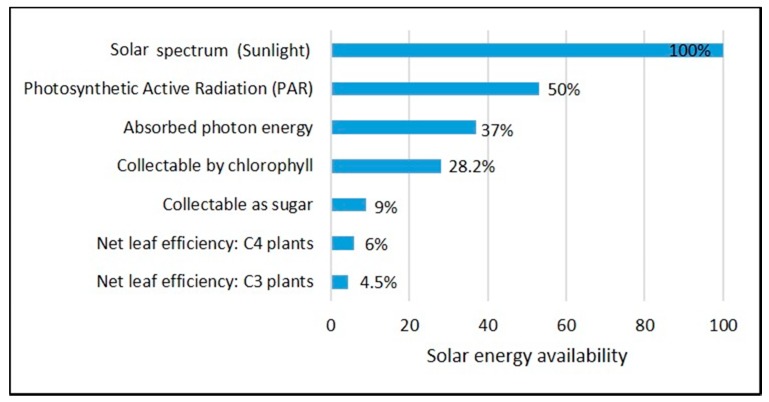
Stepwise energy loss during photosynthesis.

There are energy losses that occur from solar radiation quantum before and after the process of photosynthesis, as shown in Figure 4. Energy losses are recorded in transferring the energy to the lower frequencies and the radiation, which is available for photosynthesis called photosynthetic active radiation (PAR) that accounts for only 45–50% of energy [25]. Further on, there are also energy losses in the metabolic conversion of the products from photosynthesis, which determines the plant cells’ biochemical composition and energy yields. The oxidation state of nitrogen source (i.e., NH_4_ or NO_3_) can significantly affect biomass yield [40]. Generally, the use of NO_3_ as a nitrogen source requires extra energy and reduces biomass yield. Other factors such as concurrent respiration and photon wastage also affect photosynthetic efficiency.

The biomass yield for outdoor microalgae production can vary between 10–30% of the theoretical values. Low lipid yield from mass (outdoor) cultivation systems is considered as one of the obstacles to the viable commercial production of microalgae biodiesel [41]. Some of the factors responsible for this have been discussed as affecting photosynthesis, other inclusive factors are CO_2_ concentration, temperature, turbulence, inhibitors and contamination [31]. The flowrate of CO_2_ in a cultivation system affects pH control and biomass productivity. In assessing the effect of CO_2_ on cultivation operational conditions, it is necessary to concurrently consider carbon use efficiency, biomass productivity and the existence of pH gradients [42]. Temperature is considered to have a minimum, maximum or optimum effect on biomass production depending on the species and location. Temperature and light are considered to directly influence the microalgae growth rate [43]. Temperature is more a determinant in the efficiency of respiration in the growth of microalgae rather than a moderator. While the photochemical process is insensitive to temperature, the enzymatic process is temperature sensitive [31].

High lipid accumulation is more difficult to achieve in open ponds [41]. It is therefore necessary to close the gap between theoretical and practical solar energy conversion efficiencies. The highest reported values of conversion efficiency in C3 crops is 2.4% and for C4 crops 3.7% while short term conversion values of 3.5% and 4.3% for C3 and C4 crops respectively [44]. Insufficient capacity to utilize incident radiance lowers conversion efficiency under optimum conditions. It is only possible to achieve near theoretical results with dilute cultures and low irradiance in a shallow pond. However, mass production of microalgae implies that cultures will get optically dense, with high irradiance. Growth rate in such mass production systems is limited by the penetration of light to lower parts of the pond as a result of self-shading and absorbance of light by the water [34]. This is necessary to be considered during pond design. The implication is that while the upper part of the culture receives severe light inhibition from 90% of the absorbed solar radiance, the lower part of the pond gets the remaining portion while it is in darkness. Turbulence is often introduced to reduce this effect but it does not solve it. Dynamic light fluxes repeatedly cycled between high light levels at the culture surface and low light levels at the culture bottom in well mixed cultivation systems can also improve PCE [39]. Slowing photochemical processes or speeding up enzymatic processes are other known modifications that can improve the PCE process. Temperature regulation has also been found to improve PCE [45]. Furthermore, photoacclimation is being explored to improve light harvesting, with a significant number of research considering photoacclimation under fluctuating light, appropriate for microalgae biomass production [46].

Improving PCE to obtain higher biomass yields alone may not be the only target of a production process. High lipid yields for biodiesel production is often also desired. Although options for improving lipid yield are promising, there are significant challenges with their large-scale (outdoor) implementation [31]. Among several options for this are the selection of high lipid yield strains of microalgae species and genetic engineering to identify and improve enzymes associated with lipid production. Increasing the temporary carbon sink in biomass into storage carbohydrate rather than triacylglycerols (TAGs) improves the energy assimilation during photosynthesis, while nutrient depletion increases the lipid yield [30].

To explore the use of photosynthesis in biomass production through microalgae cultivation, the most commonly used biomass production systems in the literature are closed photobioreactors (PBRs) and open raceway ponds (ORPs) with variation or combinations of both in some cases [47]. In a bid to attain theoretical efficiency values numerous systems have been developed using the phototrophic approach. It cannot be overemphasized that cultivation must be energy efficient to be economically viable when compared with other biofuels and fossil fuels. The composition of the culture suspension, and reactor or pond geometry affect biomass yield in microalgae cultivation. The dynamic relationship between oil content and biomass growth productivity is dependent on species and physiology. The choice of a cultivation system therefore depends on the application of the biomass. Thus, there is no recommended cultivation system in the literature that has been described to fit every scenario and species [48].

ORPs are often used because of the relative low cost and energy efficient procedure of biomass production. They are commonly oval shaped with depths ranging 10–30 cm, and often equipped with paddle wheels to aid water circulation. However, ORPs have high water demands as cooling of the culture occurs via evaporation, therefore location alongside natural water sources like streams or deep sea water from oceans is advantageous [30]. Careful management (monitoring and maintenance) is required to avoid contamination, due to challenges from atmospheric exposure and open culture environment.

Closed systems on the other hand could be of algal suspensions or fixed biofilm, and they vary widely in dimensions, choice of construction materials, operating principles and design. Closed systems (bioreactors) are more commonly called photobioreactors (PBRs), as they receive light directly from the sun or other artificial light sources. They take forms of narrow tubes (horizontal or inclined), vertical coils, column structures or flat plates. Similar to ORPs, microalgae can be allowed to move freely in the growth medium but the growth medium is confined in a closed vessel, which is often transparent to allow maximum light capture. Water chemistry maintenance is easier, and the risk of contamination is limited as the exchange of liquids and gases are well controlled. However it is an energy intensive process, as liquids and gases required are introduced and removed via active pumping or bubble pressure, with an additional need for cooling. The pumping introduces turbulence and drives off oxygen, maximizing photosynthesis. PBRs, which are closed systems, present a higher complexity in terms monitoring requirements, studies that investigated these systems revealed the potential to generate large amounts of data, owing to the fast growth rate of microalgae in PBRs. Previous studies reflected the uncertainty of PBRs to be as cost competitive as ORPs in the future [34]. However, more recent studies suggest the way forward to be associated with the design and construction of PBRs from low-cost materials among several options [49]. In biofilm PBRs the microalgae are immobilized and attached to a support surface on which they grow, which simplifies the separation and recovery of the attached biomass from the culture medium [50]. The culture support surface usually consists of other microorganisms, most commonly bacteria that form the biofilm [29]. Biofilm systems could be continuously or temporarily immersed, and in other cases they could exposed to the atmosphere and only wetted by a thin water layer. Biofilm systems are known to have higher biomass productivity than suspension systems, with even much higher productivity when cultivated under mixotrophic conditions [51]. However, algal biofilm cultivation requires the use of purpose-designed systems, due to the persistent gradients in the biomass, which affects flow as well as the interaction between cells and the surface [50]. Technology has continued to emerge in the development of biofilm PBRs, with variations in horizontal and vertical static systems and biofilms PBRs with mobile support (e.g., rotating) used in the production of algal biofuels [29].

Therefore, there are several aspects of cultivation that can benefit from improved engineering, especially at large scale. Nutrient circulation and light exposure are very important and the challenge for engineers, chemists and biologists is to develop PBRs that will be low-technology based for economically viable microalgae cultivation or an efficient improvement of ORPs to optimize the use of resources with minimized tendencies of contamination [48]. A coupling of these systems could also be developed, where PBRs are used to produce pure cultures that are subsequently transferred into ORPs [52].

## 4. Microalgae Production in a Biorefinery Context

The main areas of microalgae application have been shown in Figure 1. Microalgae biomass can be used to produce a variety of biofuels and other coproducts. Microalgae can be the source of several types of biofuels, viz. methane produced during anaerobic digestion, hydrogen produced photobiologically in anaerobic conditions and biodiesel derived from lipids [53]. The most recommended environment friendly clean fuel for the transport sector is biodiesel. To obtain this from microalgae a series of treatment processes have to be applied. The complete utilization of algal biomass may involve the combination of technologies and coproduction in an integrated biorefinery approach [30].

In more recent years the biorefinery approach has become a more idealized approach for the attainment of a sustainable microalgae-based biofuels industry [30]. A biorefinery is a facility that integrates biomass conversion processes and equipment to produce a range of coproducts [54]. As the decline in petroleum prices continues to constitute an economic challenge to cost competitive biofuels production, the biorefinery approach is considered the best option for economic viability [55]. The biorefinery approach is defined as the ‘sustainable processing of biomass into a spectrum of marketable products’ [54]. In this approach the economic viability of microalgae-based biofuels is dependent on coproduction with high-value products. Previous model studies in the literature have been used to demonstrate the commercial potential of the biorefinery approach [1,33,55].

**Figure 5:**
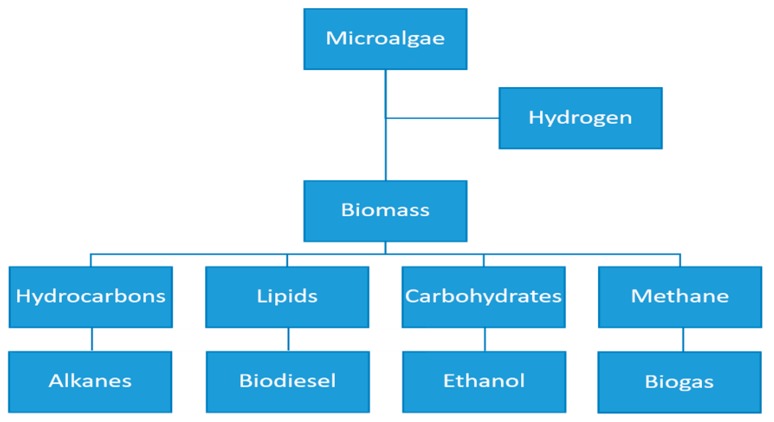
Fuel products derivable from microalgae.

While Figure 1 and Figure 5 have highlighted the possible areas of application and obtainable products from microalgae respectively, the preceding discussion has elucidated that it is possible to completely convert microalgae biomass to beneficial products. This will be a key consideration in pursuing the microalgae for biofuel agenda. In a society with depleting energy sources and increasing energy demand microalgae portends a lot of prospect as an alternative source of renewable energy, which needs to be harnessed through technological innovations.

## 5. Chemometrics as a Tool for Multivariate Analysis (MVA)

Multi-criteria decision analysis (MCDA) originated as a sub-discipline of operations research (OR) that is applied in the evaluation of multiple criteria in decision making, and it has found relevance in a wide range of areas (e.g., business and medicine) [7]. While the process that involves the extraction and correlation of physical and chemical information from an operational process to understand their effect on the operation or the outcome of the operation is called chemometrics. In chemometric analysis several non-selective signals (observations/actions) from chemical process analysis can be combined in a multivariate model to measure the desired information with high precision. In contrast to the wide scope of MCDA in OR, chemometrics is considered as a sub-discipline of chemistry. However, chemometric methods provide a backdrop on which the concepts and contributions of MCDA could be compared and understood, which can be extremely beneficial to support decision makers (DM) [5].

The ability to generate a wide range of information (i.e., multivariate data sets) from instrumental analysis requires fast computational processes in order to adequately assess such information. This requirement can be met through the use of chemometric techniques, which applies a multivariate analysis (MVA) approach [56]. This approach is able to provide elucidated information on pattern recognition through ranking, calibration, classification and prediction. Pattern recognition is the art of finding similarities, correlations and differences from measurements conducted on various samples [57]. Common pattern recognition methods include: Exploratory data analysis (e.g., principal component analysis: PCA), unsupervised techniques (e.g., cluster analysis: CA) and supervised techniques (e.g., soft independent modeling of class analogy: SIMCA) [56]. CA, PCA and discriminant analysis among other techniques can be applied to extract the most relevant information from chemical analysis of varying nature, across a range of selected criteria involved in the production process or assessment of the process [58].

In order to be applicable for general decision-making, the selected product or process criteria should aim for a holistic evaluation and to the satisfaction of certain characteristics such as relevance, completeness, non-redundancy, understandability and feasibility. They should also be clearly defined, judgmentally independent and scalable (i.e., measurable in an objective manner). The relative importance of different criteria could be modeled as weights. Excluding or including criteria, such as environmental impacts or cost, can change the outcome of MVA when chemometric methods are applied.

Chemometrics is currently being applied in chemical, biological, environmental, industrial and engineering process analysis [9]. The ability of instrumental analysis techniques such as chromatography, spectroscopy, spectrophotometry and microscopy to generate large volumes of information, even from a single chemical or environmental sample creates a need to harmonize such information into a meaningful explanation [59,60,61]. Chemometrics has been applied to a wide variety of data matrices including those collected for water [58], fungicidal [62], pharmaceutical [63], food science [64], fruits and vegetables processing [65], Parkinson’s disease [66], plant composition [67] and polymer analysis [68] studies.

## 6. Chemometrics in the Production and Processing of Microalgae

Microalgae production is a complex task as multiple criteria have to be taken into account. The complexity is due to differing perspectives, values and preferences of DMs (designers, engineers and scientists). This complexity is further compounded by the complex biology and environmental plasticity associated with microalgae species. Chemometric methods aim at supporting DMs faced with evaluating alternatives, taking into account multiple and often conflictive criteria. Multivariate modeling is achieved by measuring the desirability of achieving different levels of performance in each criteria and combining these preferences across individual criteria allowing for inter-criteria comparisons.

There are several stages involved in the production of biofuels from microalgae, which can broadly be divided into upstream and downstream phases. Microalgae species selection, geographical location and cultivation method (open or closed) can be described as the main upstream activities. While dewatering, lipid extraction, conversion and upgrading of the lipid to biofuel and further processing of the residual biomass are classified as the downstream phase. Case reviews illustrating the application of chemometrics for some of these processes from selected studies are presented and discussed. Table S1 (Supplementary Information) presents a summary of the application of chemometric methods in the production and processing of microalgae as discussed herein.

### 6.1. Characterization and Classification

There are diverse biological and inherent cellular constraints around the species production capacity of microalgae. Depending on the selected end-product strain selection and cultivation conditions will target improving the lipid or protein content of the microalgae cells [30]. Therefore, characterization is a very important process in species selection. As microalgae are a diverse group, only a small fraction of the available species form the main feedstock for biofuel production [69]. Furthermore, microalgae biomass comprises complex sets of metabolites, which makes its molecular characterization intensive [70]. The neutral lipids content of microalgae is most essential as biofuel feedstock, it is therefore important to quantify the total amount in a selected strain. Energy storage capacity of microalgae is also known to relate directly to the lipids content, with C12 to C18 chain lengths being the key ones [2,71]. Various analytical techniques are being used in this process including chromatography, electrophoresis, mass and molecular spectroscopy [72]. Mid-infra-red has been in use since the 1940s, with different techniques viz. single point, point mapping and imaging applied to study microalgae composition with mid-infrared peaks associated with proteins, lipids and carbohydrates. Inferences are drawn from qualitative observations from infra-red bands or quantitative integration and calculation of peak heights and ratios [73,74]. To assess the enormous amount data generated from processes such as microalgae cultivation and new species screening and evaluation, the combination of new methods such as infrared (IR) spectroscopy with online monitoring potential and chemometrics can contribute towards high productivity and identification. IR spectroscopy has the capacity to detect a wide range of organic compounds, with a potential for online monitoring of bioprocesses [75]. Organic molecules are known to exhibit unique signatures in the IR spectrum, especially in the mid and near IR regions [76].

In a study by Tan et al. [71] a novel cell population estimation for microalgae biomass was carried out using an unsupervised leader-follower cluster analysis on Fourier transform infra-red (FTIR) imaging of *Nannochloropsis* sp. for spectra sieving. A Varian 7000 FTIR Stingray spectrometer was used. In order to distinguish the focal plane array (FPA) pixels of microalgae cells from pixels associated with background spectra, the improved leader follower cluster analysis (iLFCA) was applied, with scripts written in Matlab^®^ v7.9. Two rounds of iLFCA were used to discriminate cell spectra from outlier counterparts. In the first round images were clustered around a user-defined number and the second round had eight clusters that well explained the measured FTIR images. The iLFCA algorithm was effective in sorting out a large number of FTIR spectra. A nominal cell size of microalgae *Nannochloropsis* sp. was compared to the spatial resolution of each FPA pixel. Subsequently, corresponding FTIR absorbance spectra was plotted together to obtain population distribution of the microalgae cells. The two iLFCA procedures took only 48 min, showing the effectiveness of the iLFCA algorithm in sorting out large number (1195) of FTIR spectra. The second study phase involved the recovery of component spectra associated with biomolecular species, using band-target entropy minimization multi-linear regression (BTEM-MLR). The component spectra from the BTEM-MLR accounted for 97% of the signal from the data set. Water, protein and lipid content of microalgae cells were evaluated on a compositional basis with this technique. Furthermore, the estimation of lipid-to-protein concentration ratio from the statistical cell population distribution was achieved. This study illustrated a technique for lipid-protein estimation statistically without the need for lipid extraction, and an additional supporting process i.e., BTEM-MLR was further used to produce concentrations and chemical maps. There are several advantages of this technique in comparison to the usual semi-quantitative methods, which use spectral peak heights or assigned wavelength to represent biomolecular constituents devoid of signals from other species. The chemometric approach relies on bilinear properties, i.e., the superpositioning principle of transmission of mid-infrared data, rather than on assumptions. The BTEM-MLR was also able to differentiate spectra contributions from major and minor comparative signals. This also significantly reduces the time taken when conducting traditional lipid-protein estimation.

In another study on classification of microalgae, Henrion et al. [77] investigated the characterization of different algae species from excitation–emission matrices (EEM) using fluorescence spectroscopy. This approach was a variation of the conventional means of characterizing phytoplankton samples by microscopic counting, which is usually time consuming. Five species representing the major phytoplankton groups (cyanobacteria, diatoms, cryptophytes, chlorophytes and crysophytes) were selected for fluorescence measurement. The EEMs covered a range of 281 excitation wavelengths and 10 emission wavelengths. The identification of the underlying spectra was achieved using PCA. A three-way PCA was applied for the identification of the underlying spectra, by decomposing the data into independent pairs of column and row profiles. Graphical representation of the first two components corresponding to emission and excitation spectrum, produced curves that served as the fingerprints for the five algae species. An advantage here was that visual comparison of the curves reveals evident differences, which gave the distinction between species. The study illustrated how a three-way PCA was used as a tool in the classification of algae species.

Traveling wave ion mobility–mass spectrometry (TWIM–MS) in combination with ultrahigh-performance liquid chromatography–high definition mass spectrometry (UHPLC-HDMS) has also been applied for the characterization of microalgae using data-independent analysis [70]. Variant cultivation times, culture media composition and light exposure were combined to produce 16 sample types from across five generic species of microalgae viz. *Tetraselmis* sp., *Spirulina* sp., *Dunaliella* sp., *Chlorella* sp. and *Scenedesmus* sp. The process involved the extraction of lyophilized biomass from the microalgae samples using the Blight and Dyer-like method. TWIM–MS was performed by direct infusion and UHPLC-HDMS acquisition was performed in ion-mobility mode. Prior to the data analysis spectra normalization and Pareto scaling were applied as preprocessing. Progenesis QI Informatics software was used for data analysis. From the 16 analyzed samples, it was possible to annotate 1251 compounds, with 210 classified as lipids. The approach applied in this study demonstrated the efficiency of the statistical and binary comparison methods for screening lipids and metabolites. The analysis strategy explored in this work offers a powerful tool for the microalgae industry by aiding in the identification of ideal strains and culture conditions for application in the production of biofuels, saving analysis time and facilitating identification of a large number of constituents at once.

The discussed techniques can be effectively applied to select microalgae species from environmental samples and also to screen suitable phenotypes with high neutral lipid suitable for biofuel production from a large number of samples, within a reasonably short time.

### 6.2. Upstream Processes

Microalgae cultivation must be economically advantageous and energy efficient to reach the scale of biomass commensurate with other biofuels [30]. The cultivation of microalgae is mainly done in PBRs, ORPs or a combination of the two. It is also important for photosynthesis to be efficient in the selected process with natural sunlight or an artificial source of light. These considerations come to bear on the design of cultivation systems, as well as other cultivation conditions. Chemometrics has played a role in establishing relationships in the selection criteria and how that can affect system performance in processes across several disciplines [78].

#### 6.2.1. Cultivation System Selection and Operation

In the design of cultivation systems it is often the practice to develop systems that can be operated on a bench scale and subsequently increase it to a pilot scale. The challenge however is that performance often varies on scale up, thus requiring a reassessment or complete change considering several factors involved in the process. The engineering design principles applied in scaling up can also be used in scaling down. Tescoine et al. [79] applied the principal component analysis (PCA) in the design of a cell culture bioreactor. The production process was performed at a 200 L for prototyping and 2000 L for manufacturing. The variable factors considered were the temperature, pH, seed density, medium formulation, feed amount, nutrient feed formulation, culture duration and agitation rate. The culture was sampled at regular frequencies to monitor the process performance. Cell culture data were compiled in Microsoft Excel and imported to SIMCA-P+ for both manufacturing and pilot scale bioreactors. The data was normalized by applying unit variance scaling and mean centering to all variables. MVA was used to supplement univariate analysis, and the PCA was useful for process trouble shooting and comparison between data sets [80]. PCA module was built using four principal components that represented 85% of the initial data set. Subsequently, the product quality was generated and verified following the model. The verified results fell within 95% confidence limit of the model and the approach used can serve as a model development procedure for cell culture processes.

San Pedro et al. [81,82,83] investigated the cultivation of the microalgae *Nannochloropsis gaditana* using ORP, PBR and flat-panel bioreactor (FPB) in outdoor pilot scale studies. The system performance in each case was assessed through culture parameter, growth modeling and lipid productivity investigations. Parameters considered to affect lipid productivity were dilution rate of nutrient supply (N and P), irradiance as a measure of light availability and temperature. These parameters were applied to develop models used in describing *Nannochloropsis gaditana* biomass productivity and lipid yield. The models were considered a powerful tool for the improvement and scale-up of microalgae production for biofuels.

In the study that investigated ORPs [81], *Nannochloropsis gaditana* was cultivated in three pilot scale ORPs (7.2 m^2^), equipped with electrically driven paddle wheels. Solar radiation, CO_2_ injection rate, temperature, pH and dissolved oxygen (DO) were monitored using a data logging system and control software. Equations were developed for the growth model and metabolite production of *Nannochloropsis gaditana* measured in the cultivation process. Dilution rate was varied in the range 0.3–0.6 L/day with a culture medium containing 10.5 mM NO_3_^−^ and 0.8 mM PO_4_^2−^. An optimum biomass productivity of 0.19 L/day was achieved at a dilution rate of 0.3 L/day, temperature of 29 ± 2.5 °C and irradiance of 2105 ± 55 µE/m^2^.s. The results showed that fatty acid (FA) and lipid contents were inversely related to the dilution rate. The combination of lower dilution rates, lower-average irradiance and high temperature within the study range resulted in 12% and 26% FA and total lipids respectively.

For the PBR system [82], different culture conditions were applied in a reactor of 340 L volume with vertical solar receivers arranged to optimize solar radiation capture, with a similar data logging system with the ORP [81]. A key consideration factor for outdoor cultivation systems is the optimal dilution rate to optimize biomass/lipid productivity. Furthermore, volume to surface ratio should also be effectively optimized to improve areal productivity of cultivation systems. The culture medium contained 10.4 mM NO_3_^−^ and 0.8 mM PO_4_^2−^, a dilution range of 0.1–0.6 L/day, solar irradiance of up to 2100 µE/m^2^.s and temperature 25–30 °C were investigated. Optimum biomass productivity of 0.59 g/L was found in a dilution range of 0.31–0.35 g/L, with irradiance of 475 µE/m^2^.s. An increase in protein content was observed with higher dilution rates, indicating nitrogen conversion to proteins. FA and lipid contents decreased with dilution rate increase, indicating no accumulation enhancement occurred because maximal biomass productivity was the same as maximum for FA and lipid compounds.

In the investigation with the FPB system [83], disposable plastic bags of 1.7 m by 2.5 m dimensions, hanged in steel frames were used. Dilution rate of 0.1–0.4 L/day with 10 mM NO_3_^−^ and 0.8 mM PO_4_^2−^ were applied. These studies allowed the establishment of the influence of culture conditions and reactor design and orientation on *Nannochloropsis gaditana* productivity. From the three cultivation systems it was possible to optimize growth conditions in terms of the culture medium, temperature and dilution rate with *Nannochloropsis gaditana* using the developed models. The importance of the models lied in their reliability and ease of use for calculating large-scale biofuel requirements when considering the productivity of microalgae, especially in terms of lipid production.

It is often a challenge to produce representative bioreactor models at scale up when comparing with pilot scale. Previous studies have illustrated the use of two components, which includes the experimental testing of the scaling criteria and applying statistical assessment to optimize scale-up of microalgae cultivation. These studies were representative to qualify the process in terms of productivity and product quality. As illustrated in the foregoing discussions, there are several other factors in addition to the choice of a cultivation system, which affect microalgae productivity during cultivation. These factors cut across system, culture and environmental conditions, with some prominent ones including cell physiology, growth phase, temperature, CO_2_ concentration, light availability and culture medium composition. The application of various chemometric methods in assessing these other factors were further presented.

An assessment of cell physiology in all stages of microalgae growth under cultivation plays an important role for environmental and ecological monitoring purposes [84]. In order to maximize lipid production in the cultivation of microalgae a controlled open or closed system is also necessary [85]. Several factors affect the optimum growth and lipid accumulation of microalgae in controlled systems, including temperature, pH and the availability and concentration of macro- and micro-nutrients [2]. Conventional laboratory techniques of investigating these factors are labor intensive and time consuming.

Growth phases of microalgae were monitored using Raman spectroscopy in a study, which investigated two freshwater species viz. *Microcystis flos-aquae* and *Chlorella vulgaris* [84]. The microalgae cells were adhered to glass slides and coated with poly-l-lysine, then Raman mapping with the aid of a microscope was conducted. Representative spectra of each algal cell were chosen in a mapping data set. Correlation of Raman analysis of the two microalgae species and the characteristic Raman intensities were fitted to gather statistical information used in assessing growth phases and physiological responses. Raman spectra of algal cells induced with different environmental conditions were classified by their PCA scores, in a process that applied dimensionality reduction prior to MVA. Subsequently, support vector machine (SVM), which is a machine learning method was applied to identify growth phases under different culture conditions. Raman characteristic peaks under different environmental conditions were mined from the daily spectra of microalgae, and band assignment was applied. Classification of culture conditions was 65% accurate. Although samples were used to construct models for the identification and classification of culture conditions with PCA and SVM, Raman spectra was also found applicable for the assessment of environmental conditions in microalgae cultivation. The results demonstrated that the combination of PCA and SVM was not only useful in assessing cultivation but also predicted the preliminary environmental condition of microalgae in water bodies, which is useful to check eutrophication.

Jiménez et al. [86] studied the relationship between physicochemical variables and productivity of *Spirulina* sp. in open ponds. PCA was used to assess different sets of samples characterized by a combination of temperature, DO concentration, pH, biomass productivity, irradiance and conductivity, with 240 cases included in the analysis. From the results it was deduced that pH and salinity led to loss in productivity in open ponds around mid-summer, incidence with high rates of dissolved oxygen. The application of this method aided the prediction of significant loss of productivity in the open ponds, resulting from high pH and high-dissolved O_2_ concentration during summer. This information can guide in selecting the best cultivation periods or the need to include other measures under such conditions.

#### 6.2.2. Nutrient Sources and Conditions

The investigation of alternative nutrient sources such as agricultural wastewater and farmyard compost are also being increasingly explored for microalgae cultivation. Chicken compost was investigated as a source of nutrients in the cultivation of *Chlorella vulgaris* and its effect on growth and fatty acid composition was assessed [87]. Parameters considered were culture medium nutrient addition, volume and circulation time (duration till removal). The specific growth rate and biomass productivity were the main performance indicators. Growth was consistent with an optimum growth rate of 0.6 day^−1^ and biomass concentration of 0.8 g/L. Optimum production conditions were: 30% (*v/v*) removal of cultivation medium and 0.04 L/L of chicken compost in a cultivation medium of pH 3. High nutrient concentration had negatively affected biomass productivity. Overall lipid content was found in the range of 26–37%, with an average of 31%. In this study chicken compost was found to be a rich alternative source of nutrients in the production of microalgae.

Corresponding to many factors imbibed, nitrogen among several nutrients plays a key role in the macromolecular synthesis pathway in microorganisms including microalgae [88]. In a study which investigated the effect of different nitrogen sources on lipid content in microalgae, chemometric analysis was applied to analyze FTIR spectral data [89]. *Chloromonas* sp. was cultivated using four different nitrogen sources viz. urea, sodium nitrate, ammonium nitrate and potassium nitrate. SAS JMP software was used to analyze data from two FTIR spectral regions i.e., lipid acyl and biomolecular fingerprint regions in a PCA and multidimensional scaling (MDS) assessment. PC1 and PC2 accounted for 98.79% of variability in the lipid acyl profiles region (i.e., 2800–3000 cm^−1^) and 94.86% of the variability in the biomolecular fingerprint regions (i.e., 1000–1800 cm^−1^). The results indicated that in a preferential order urea  >  sodium nitrate  >  ammonium nitrate  >  potassium nitrate, had the most significant effects on lipid yield. MVA of the FTIR spectra demonstrated the applicability of the approach as a useful technique for rapid assessment of microalgae production process.

The effect of nitrogen stress on lipids concentration was also investigated by Li et al. [90]. *S. obliquus* cells were inoculated under three nitrogen treatments, with concentrations of 0, 0.3 and 1.5 g/L NaNO_3_ representing nitrogen-depleted, nitrogen-deficient and nitrogen-adequate conditions. Total lipids and dry substance content were determined using gravimetric methods. Raman spectra of 63 microalgae samples was collected and subsequently correlated with the total lipid contents in a multivariate linear regression model using Matlab R2016a. For Raman imaging, a pixel-by-pixel data cube image was imported from the quantitative determination model. From the results, it was observed that faster lipid accumulation was achieved in the first four days under stress with increase in stress intensity. Using this approach, a quantitative visualization of intracellular lipids concentration in microalgae cells was achieved, effectively providing spatial and temporal dynamic characteristics of the intracellular lipids of the microalgae cells under nitrogen stress. Revealing metabolism information of intracellular lipid accumulation can effectively enhance the production of microalgae biomass for biofuels production.

#### 6.2.3. Photoperiod and Trophic Conditions

Light is one of the most important environmental factors that affect chlorophyll synthesis and other plant growth aspects, and it is also used as an environmental signal for the control of morphogenetic processes [91,92]. There are several means of investigating the effect of light and dark cycles in the production of microalgae under three main trophic conditions viz. autotrophic, mixotrophic and heterotrophic conditions [93]. There are some species of microalgae that are able to switch between photo- and heterotrophic metabolisms. However, light has multiple effects on photosynthetic organisms by providing energy as well as signals that influence the composition of intracellular components [94].

Matos et al. [93] investigated the cultivation of *Nannochloropsis gaditana*. The analysis was performed by a one-way analysis of variance (ANOVA) using STATISTICA version 7.0. The study was conducted under five desalination concentrations (DC), four photoperiod schedules and three trophic conditions. Considering the large number of variables involved, the amount of biomass, lipid/protein contents and fatty acids produced under different phototrophic and photoperiod regimes were selected for PCA. PC1 and PC2 represented 63.38% of the total variability. Cluster A captured the main fatty acids under heterotrophic conditions, under mixotrophic conditions increased protein levels were associated with cluster C, while cluster D was positively associated with lipids, while monounsaturated fatty acids (MUFA) and only one fatty acid were in cluster B. This study and the statistical analysis revealed that by varying growth conditions the chemical composition of microalgae could be manipulated. This again will be useful when targeting a specified end product.

#### 6.2.4. Proteomic Metabolism

The use of mass spectroscopy is commonly applied in proteomic methodologies, with the capacity to generate a large amount of data [95]. The growth and physiology of microalgae are affected by various metabolic processes, which determines cellular abundance, biometry and micromorphology, as well as lipid production [96]. Lipids are known to contribute to the modulation of cell function [97]. Modulation of lipid biosynthesis could also be attained through membrane reorganization or degradation [98].

Gérin et al. [99] studied proteomic adaptations in *Chlamydomonas reinhardtii* induced by light, carbon and inorganic nitrogen. The statistical design of experiment (DOE) comprised 32 culture conditions. Relative protein abundance was quantified by two dimensional differential in-gel electrophoresis (2D-DIGE). A hierarchical clustering enabled partitioning of the biological variables into eight clusters. The DOE was carried out using JMP 11 software (SAS). Partial least square regression (PLSR) was run in the multivariate methods platform with leave out one validation and multi-linear regression (MLR) was applied in the fit model platform, then PCA was subsequently performed. Environmental variables were also taken into consideration in addition to biological variables. Regulation of the primary metabolism was overall shown to be a multifactorial issue, as there were complex superimpositions that influenced nearly all the biological variables. The authors concluded that combining this method with other omics methods could improve current understanding of systems biology in diverse organisms.

### 6.3. Downstream Processes

The downstream processes of microalgae based biofuel production have in recent times gained a lot of attention. This has translated into technological advances, increasing the production on commercial scales. However, present production costs do not allow microalgae-based biofuels to favorably compete with fossil-based fuels. Through the use of chemometrics it is possible to gain additional knowledge on various interrelated processes, which can lead to a harmonized integration of the lipid extraction, conversion and upgrading processes, and in turn increase the efficiency of the entire process.

#### 6.3.1. Dewatering

Dewatering constitutes a major bottleneck in the production of microalgae, especially due to high energy requirements. Several dewatering approaches have been explored at laboratory, pilot and commercial scales [100,101,102,103]. However, there remains a need for energy efficient techniques that can lead to large scale processing of microalgae for biofuels [104].

There was no chemometric study on microalgae dewatering found in the literature. The chemometric analysis from an unpublished study by the current authors is presented [105]. The dewatering of microalgae in a flocculant assisted dynamic filtration process was explored using the Britt dynamic drainage jar (BDDJ). In this study, the performance of 12 different flocculants was investigated for the dewatering of microalgae *Dictyosphaerium* sp. The effects flocculant dose, pH, shear, zeta potential, flocculant molecular weight and flowrate on the efficiency of the process was considered. The sequential chemometric analysis of 209 observations across varying system conditions was assessed using the preference ranking organization method for enrichment evaluation (PROMETHEE) and graphical analysis for interactive aid (GAIA). This assessment was able to provide a complete ranking of the flocculants based on their outranking flow.

PROMETHEE and GAIA is capable of assessing multiple criteria holistically through pair wise comparison, rank the investigated flocculants and identify the most promising options among the flocculants. Furthermore, it allows for a graphic representation of the criteria using GAIA, which provides a better understanding of the inter-dimensional interactions and conflicts of criteria, thereby facilitating consensus building in decision-making processes.

#### 6.3.2. Lipid Disruption, Extraction and Estimation

There are several methods for lipid extraction from microalgae, with the most common ones being Sohxlet, Folch and Bligh and Dyer methods [106]. These methods involve the use of organic solvents (e.g., hexane, chloroform and methanol). Although these methods have been found to be very effective, the use of organic solvents of fossil origin is an issue of concern, due to its negative environmental and health concerns [107]. There is an ongoing search for alternative extraction methods, which are considered to be safe to both the environment and human health. These solvents are being developed on the principles of green chemistry and are referred to as ‘green solvents’. However, there is limited information on the performance of this green solvents in comparison with traditional solvents [106]. Recent studies in microalgae employ extraction strategies that involve the selection of improved formula for solvents, as well as other technologies like supercritical fluid extraction and electrical disruption, which do not use toxic solvents [108].

Ma et al. [108] optimized a microalgae cell disruption technique using nuclear magnetic resonance (NMR) coupled with chemometrics. Three species of microalgae were used in this study, and NMR was used for the simultaneous identification and quantification of metabolites. The gravimetric analysis of response variables was performed in GraphPad Prism and crosschecked with SPSS. All variables were log-centered and scaled to unit variance. The identification of metabolites from a high complexity of microalgae extract was achieved in this study. Furthermore, the PCA and partial list squares discriminant analysis (PLSDA) revealed the effect of pre-treatment.

*Botryococcus braunii* was used in biofuels production by Talukdar et al. [109]. Elemental composition and higher heating value (HHV) were assessed for samples extracted using wet solvent extraction processes. The experimental data was evaluated using one-way ANOVA. The hierarchical cluster dendograms for the FTIR spectra were plotted with an aim to assign spectral variability to chemical heterogeneity. The PCA revealed the most prominent variation patterns. The first two PCs accounted for 94.34% of variability in the data set. In this study ATR-FTIR combined with chemometrics revealed related spectra patterns in the data set. The study illustrated the efficiency of high lipid extraction with the support of statistical tools.

#### 6.3.3. Hydrothermal Liquefaction (HTL)

HTL is a viable route used in the conversion of a wide range of feedstock to liquid fuels [110]. Most HTL techniques for biodiesel production currently deployed fall short of the diesel standard [111]. Therefore, there is an opportunity to improve the quality of RDB produced via HTL using chemometric techniques.

Madsen et al. [112] analyzed the composition of biocrude from hydrothermal liquefaction. PCA was performed with Matlab using PLS Toolbox 8.0.1. PCA did not separate the thermally and non-thermally treated samples. This indicated that the pretreatment methods only increase dispersibility of the feedstock without altering the polymeric material. The hierarchical clustering was illustrated with K-nearest neighbors (kNN) classification.

#### 6.3.4. Fuel Quantity and Quality Estimation

Several strategies have been developed to modify the properties of RDB through solvent extraction, catalytic cracking, distillation, etc. [111,113]. These strategies are applied in the modification of properties such as the chemical composition, heating value, nitrogen, sulphur and oxygen contents, viscosity and density [111].

Nascimento et al. [114] studied the productivity and quality estimation of microalgae biodiesel. Two species of microalgae were considered, viz. *Chlamydomonas* sp. and *Scenedesmus obliquus*. CA was performed with R Package vegan 2.1–3, while PCA was performed with Canoco 4.5^®^. The first and the second axis of the PCA explained 56.8% and 23.4% of the observed variations respectively. The study suggested that the criteria for strain selection could improve the algae-based biodiesel industry.

In another study, Islam et al. [115] investigated the influence of structural features of fatty acid on the physical and chemical properties of biodiesel. The fatty acid structural features considered were the chain length, degree of unsaturation and branching of the carbon chain. The fatty acid profiles were used in species selection and biodiesel characterization, from nine species of microalgae used in the study and another 12 from the literature. An equal parameter ranking was performed using PROMETHEE and GAIA. *Nanochloropsis occulata*, *Extubocellulus* sp. and *Biddulphia* sp. were found to be the only species from the studied samples, which met EN 14214 and ASTM D6751–2 diesel standards. The recommended that for biodiesel production, it is better to survey a large number of algal species and optimize the desired conditions to attain maximum productivity [2]. In the bid to achieve this feat, chemometrics will be a useful tool.

## 7. Concluding Remarks and Future Perspectives

To fully exploit the derivable benefits from microalgae, a better understanding of the parameters influencing the growth rate, biomass and lipid accumulation is critical to optimize the productivity. The selection and improvement of high yield species, design and construction of low cost and low energy demand cultivation systems, efficient dewatering techniques, non-chemical extraction methods and wet conversion processes are required to improve sustainability of the microalgae industry.

The use of chemometrics through various stages of microalgae production and processing has been illustrated through the cases discussed. The application of chemometrics using MVA techniques like PCA holds a promising prospect for a wide range of applications, especially in the production of microalgae [116]. In order to enhance the microalgae production process to a level where it can be economically deployed in the production of biofuels, chemometrics can make potential contributions to the process optimization. Chemometrics offers valuable insight on the contribution of a range of parameters to the performance of a system. This methodology could be performed in early selection stages and can guide DMs to select the most appropriate process. The ability to effectively apply this technique is not only time saving, but also has beneficial financial impacts.

Large-scale production of microalgae for low value products like biofuels is still hindered by unique challenges at various stages along the production process chain. Despite these challenges, the potential for improved productivity at lower costs are high. The application of online measurement systems coupled with chemometric analysis in large-scale microalgae production within the biorefinery context, will be an exciting development that will unfold within the next few years. Microalgae production also has other potential socioeconomic and environmental benefits that will sustain the growth of the industry, as the biofuel technology continues to develop.

## Supplementary Materials

The following are available online at https://www.mdpi.com/2073-4409/8/8/851/s1, Table S1: Summary of chemometric methods applied in the production and processing of microalgae for biofuels.

## Abbreviations

2D-DIGE	Two Dimensional Differential In-Gel Electrophoresis
ANOVA	Analysis of Variance
BDDJ	Britt Dynamic Drainage Jar
BEV	Battery Electric Vehicle
BTEM-MLR	Band-Target Entropy Minimization Multi-Linear Regression
CA	Cluster Analysis
DC	Desalination Concentration
DM	Decision Maker
DO	Dissolved Oxygen
DOE	Design of Experiment
EEM	Excitation-Emission Matrices
FA	Fatty Acid
FAME	Fatty Acid Methyl Ester
FCV	Fuel Cell Vehicle
FPA	Focal Plane Array
FPB	Flat Plate Bioreactor
FTIR	Fourier Transform Infra-Red
GAIA	Graphical Analysis for Interactive Aid
GC	Gas Chromatography
GC-FID	Gas Chromatography-Flame Ionization Detection
GC-MS	Gas Chromatography-Mass Spectrometry
GHG	Greenhouse Gas
HHV	Higher Heating Value
iLFCA	Improved Leader Follower Cluster Analysis
IR	Infra-Red
kNN	k-Nearest Neighbors
MCDA	Multi-Criteria Decision Analysis
MDS	Multi-Dimensional Scaling
MLR	Multi-Linear Regression
MUFA	Monounsaturated Fatty Acid
MVA	Multi-Variate Analysis
NMR	Nuclear Magnetic Resonance
NREL	National Renewable Energy Laboratory
OFAT	One Factor At a Time
OR	Operations Research
ORP	Open Raceway Pond
PAR	Photosynthetic Active Radiation
PBR	Photobioreactor
PCA	Principal Component Analysis
PCE	Photon Conversion Efficiency
PHEV	Plug-in Hybrid Electric Vehicle
PLSDA	Partial List Squares Discriminant Analysis
PLSR	Partial Least Square Regression
PROMETHEE	Preference Ranking Organization Method for Enrichment Evaluation
RDB	Renewable Diesel Blendstock
SIMCA	Soft Independent Modeling of Class Analogy
SVM	Support Vector Machine
TAG	Triacylglycerol
TAPPI	Technical Association of the Pulp and Paper Industry
TWIM–MS	Traveling Wave Ion Mobility–Mass Spectrometry
UHPLC–HDMS	Ultrahigh-Performance Liquid Chromatography–High Definition Mass Spectrometry
