# Supplementary material for: Factors Affecting Microalgae Production for Biofuels and the Potentials of Chemometric Methods in Assessing and Optimizing Productivity
Source: Cells. 2019 Aug 7;8(8):851. doi: 10.3390/cells8080851 (PMC6721732; doi:10.3390/cells8080851)
Supplement: Supplementary file 1 [file cells-08-00851-s001.pdf]

Factors affecting microalgae production for biofuels and the potentials of chemometric methods in assessing and optimizing productivity

Mutah Musa [1,*], Godwin A. Ayoko [2], Andrew Ward [3,4], Christine Rösch [5], Richard J. Brown [1] and Thomas J. Rainey [1,*]

[1] Biofuel Engine Research Facility, School of Chemistry, Physics and Mechanical Engineering, Science and Engineering Faculty, Queensland University of Technology (QUT), Queensland 4000, Australia; Emails: ml.musa@qut.edu.au (M.M.); richard.brown@qut.edu.au (R.J.B.); t.rainey@qut.edu.au (T.J.R.)

[2] Environmental Technologies Discipline, School of Chemistry, Physics and Mechanical Engineering, Science and Engineering Faculty, Queensland University of Technology, Queensland 4000, Australia; Email: g.ayoko@qut.edu.au (G.A.A.)

[3] Queensland Urban Utilities (QUU), Innovation Centre, Main Beach Road Myrtletown QLD 4008, Australia; Email: andrew.ward@urbanutilities.com.au (A.W.)

[4] Advanced Water Management Centre (AWMC), University of Queensland (UQ), St Lucia, Brisbane, Queensland, 4072, Australia (A.W.)

[5] Institute for Technology Assessment and Systems Analysis (ITAS), Karlsruhe Institute of Technology, 76021 Karlsruhe, Germany (C.R.)

* Corresponding authors.

Supplementary information

Table S1: Summary of chemometric methods applied in the production and processing of microalgae for biofuels.

**Study focus**	**Parameters considered**	**Analytical method**	**Chemometric method applied**	**Software applied**	**Reference**
Cell population estimation	Cell growth rate, light intensity & air flowrate	FTIR spectroscopy and microscopy	iLFCA, BTEM-MLR	Matlab v7.9	[1]
Classification	EEM of fluorescence intensity	Fluorescence spectroscopy	PCA	-	[2]
Characterization	Cell lipid fraction	TWIM-MS and UHPLC-HDMS	PCA	EZinfo 2.0v & Progenesis QI Informatics software	[3]
Bioreactor design	Temperature, pH, feed amount, agitation rate & seed density	Ion chromatography and fluorescence detection.	PCA	MS-Excel and SIMCA-P+	[4]
Outdoor production (cultivation)	Biomass concentration, irradiance, nutrient	UV/Vis Spectrophotometry, fluorescence spectroscopy,	One-way and multivariate analysis	Statgraphics Centurion XVI software	[5–7]

using ORP, PBR and FPB	supply: nitrate and phosphate input & dilution rate	centrifugation, freeze drying, GC-MS and microscopy			
Growth phase monitoring	N_2 concentration, light flux and cell growth rate	Raman spectroscopy and microscopy	PCA and SVM	Matlab R2013a	[8]
Effect of physicochemical properties on ORP productivity	Temperature, DO concentration, pH, irradiance, conductivity & biomass productivity	Dosimetry, culture circulation speed, pH/OXI meter readings	PCA	-	[9]
Alternative nutrient source (chicken compost)	Nutrient addition rate, volume, circulation time, specific growth rate and biomass productivity	UV spectrophotometry, centrifugation, GC, FTIR spectroscopy & GC-FID	ANOVA followed by pairwise comparison	SPSS	[10]
Effect of N_2 source on lipid content	N_2 sources ($CO(NH_2)_2$, NH_4NO_3 & KNO_3) cocentration, specific growth rate	FTIR spectroscopy, gravimetric determination of biomass & lipid content after drying & extraction respectively	CA, MDS & PCA	SAS JMP software	[11]
Effect of N_2 stress	Adequate, deficient & depleted N_2 conditions, light intensity, photoperiod & cell count	Gravimetric lipid estimation & Raman micro-spectroscopy	PLSR	Matlab R2016a	[12]
Effect of photoperiod & trophic conditions	Auto-, mixo- & heterotrophic conditions, DC, photoperiod regime, conductivity, light intensity & growth phase	Cytometry, microscopy, centrifugation, gravimetric biomass determination & GC	One-way ANOVA followed by pairwise Tukey test & PCA	STATISTICA v7.0	[13]
Proteomic adaptation	Light intensity, CO_2, NO_3 & NH_4 concentrations,	2D-DIGE, spectrophotometry & fluorimetry	MLR, PLSR & PCA	JMP 11 software (SAS)	[14]

	relative protein abundance				
Dilute suspension dewatering	Flocculant dose, pH, stirring rate, zeta potential, flowrate & biomass retention efficiency	UV/Vis spectroscopy, gravimetric biomass determination, Rheology, FTIR spectroscopy & zeta potential measurements	PROMETHEE & GAIA (outranking flow)	DecisionLab 2000	[15]
Cell disruption	Extraction method (homogenizing, cryogrinding, sonication & HCl extraction) & yield of bioactive metabolites	Gravimetric analysis & NMR	Two-way ANOVA with Tukey's test, PCA & PLSDA	GraphPad Prism & SPSS	[16]
In-situ wet process solvent extraction	Elemental composition, biomass & ash content, HHV	FTIR, microscopy, UV/Vis spectrophotometry, gravimetric biomass determination & GC-MS	One-way ANOVA & PCA	Graph Pad Prism 5.0	[17]
Biocrude composition analysis	Thermal & non-thermal pretreatment	GC-MS & thermogravimetric analysis	PCA & CA using kNN classification	Matlab	[18]
Lipid productivity & fuel quality estimation	FAME profiles, cetane number, iodine value & oxidation stability	UV/Vis spectrophotometer, GC	PCA & CA	R Package vegan 2.1–3 & Canoco 4.5®	[19]
Influence of fatty acid features on biodiesel properties	Cetane number, iodine value, density, kinematic viscosity & HHV	GC-MS, GC-FID, fuel property estimation from FAME profiles	PROMETHEE & GAIA	DecisionLab 2000	[20]

- the full meaning of all abbreviations used in Table S1 have been provided in at the first mention of each abbreviation within the article and in the table of abbreviations included at the end of the article.

References

1. Tan, S. T., Balasubramanian, R. K., Das, P., Obbard, J. P., Chew, W. Application of mid-infrared chemical imaging and multivariate chemometrics analyses to characterise a population of microalgae cells. *Bioresour. Technol.* **2013**, *134*, 316–323. doi:10.1016/j.biortech.2013.01.060.

2. Henrion, R., Henrion, G., Böhme, M., Behrendt, H. Three-way Principal Components Analysis for fluorescence spectroscopic classification of algae species. *Fresenius. J. Anal. Chem.* **1997**, *357*, 522–526. doi:10.1007/s002160050206.

3. Fasciotti, M., Souza, G. H. M. F., Astarita, G., Costa, I. C. R., Monteiro, T. V. C., Teixeira, C. M. L. L., Eberlin, M. N., Sarpal, A. S. Investigating the Potential of Ion Mobility-Mass Spectrometry for Microalgae Biomass Characterization. *Anal. Chem.* **2019**, acs.analchem.9b02172. doi:10.1021/acs.analchem.9b02172.

4. Tescione, L., Lambropoulos, J., Paranandi, M. R., Makagiansar, H., Ryll, T. Application of bioreactor design principles and multivariate analysis for development of cell culture scale down models. *Biotechnol. Bioeng.* **2015**, *112* (1), 84–97. doi:10.1002/bit.25330.

5. San Pedro, A., González-López, C. V. V., Acién, F. G. G., Molina-Grima, E. Outdoor pilot production of Nannochloropsis gaditana: Influence of culture parameters and lipid production rates in flat-panel photobioreactors. *Algal Res.* **2016**, *18*, 156–165.

6. San Pedro, A., González-López, C. V., Acién, F. G., Molina-Grima, E. Outdoor pilot production of Nannochloropsis gaditana: Influence of culture parameters and lipid production rates in raceway ponds. *Algal Res.* **2015**, *8*, 205–213. doi:10.1016/J.ALGAL.2015.02.013.

7. San Pedro, A., González-López, C. V., Acién, F. G., Molina-Grima, E. Outdoor pilot-scale production of Nannochloropsis gaditana: Influence of culture parameters and lipid production rates in tubular photobioreactors. *Bioresour. Technol.* **2014**, *169*, 667–676. doi:10.1016/J.BIORTECH.2014.07.052.

8. He, S., Fang, S., Xie, W., Zhang, P., Li, Z., Zhou, D., Zhang, Z., Guo, J., Du, C., Du, J., Wang, D. Assessment of physiological responses and growth phases of different microalgae under environmental changes by Raman spectroscopy with chemometrics. *Spectrochim. Acta - Part A Mol. Biomol. Spectrosc.* **2018**, *204*, 287–294. doi:10.1016/j.saa.2018.06.060.

9. Jiménez, C., Cossío, B. R., Niell, F. X. Relationship between physicochemical variables and productivity in open ponds for the production of Spirulina: A predictive model of algal yield. *Aquaculture* **2003**, *221* (1–4), 331–345. doi:10.1016/S0044-8486(03)00123-6.

10. Tan, X. B., Lam, M. K., Uemura, Y., Lim, J. W., Wong, C. Y., Ramli, A., Kiew, P. L., Lee, K. T. Semi-continuous cultivation of Chlorella vulgaris using chicken compost as nutrients source: Growth optimization study and fatty acid composition analysis. *Energy Convers. Manag.* **2018**, *164*, 363–373. doi:10.1016/j.enconman.2018.03.020.

11. Difusa, A., Mohanty, K., Goud, V. V. The chemometric approach applied to FTIR spectral data for the analysis of lipid content in microalgae cultivated in different nitrogen sources. *Biomass Convers. Biorefinery* **2016**, *6* (4), 427–433. doi:10.1007/s13399-016-0198-6.

12. Li, X., Sha, J., Chu, B., Wei, Y., Huang, W., Zhou, H., Xu, N., He, Y. Quantitative visualization of intracellular lipids concentration in a microalgae cell based on Raman micro-spectroscopy coupled with chemometrics. *Sensors Actuators B Chem.* **2019**, *292*, 7–15. doi:10.1016/J.SNB.2019.04.048.

13. Matos, Â. P., Cavanholi, M. G., Moecke, E. H. S., Sant'Anna, E. S. Effects of different photoperiod and trophic conditions on biomass, protein and lipid production by the marine alga Nannochloropsis gaditana at optimal concentration of desalination concentrate. *Bioresour. Technol.* **2016**, *224*, 490–497. doi:10.1016/j.biortech.2016.11.004.

14. Gérin, S., Leprince, P., Sluse, F. E., Franck, F., Mathy, G. New Features on the Environmental Regulation of Metabolism Revealed by Modeling the Cellular Proteomic Adaptations Induced by Light, Carbon, and Inorganic Nitrogen in Chlamydomonas reinhardtii. *Front. Plant Sci.* **2016**, *7* (August), 1158. doi:10.3389/fpls.2016.01158.

15. Musa, M., Ward, A., Ayoko, G. A., Rösch, C., Brown, R., Rainey, T. J. New approach to single-step dynamic dewatering of microalgae from dilute suspensions. *(unpublished)* **2019**.

16. Ma, N. L., Teh, K. Y., Lam, S. S., Kaben, A. M., Cha, T. S. Optimization of cell disruption methods for efficient recovery of bioactive metabolites via NMR of three freshwater microalgae (chlorophyta). *Bioresour. Technol.* **2015**, *190*, 536–542. doi:10.1016/j.biortech.2015.03.036.

17. Talukdar, J., Kalita, M. C., Goswami, B. C., Hong, D. D., Das, H. C. Liquid Hydrocarbon Production Potential of a Novel Strain of the Microalga *Botryococcus braunii* : Assessing the Reliability of in Situ Hydrocarbon Recovery by Wet Process Solvent Extraction. *Energy & Fuels* **2014**, *28* (6), 3747–3758.

doi:10.1021/ef402298r.

18. Madsen, R. B., Lappa, E., Christensen, P. S., Jensen, M. M., Klemmer, M., Becker, J., Iversen, B. B., Glasius, M. Chemometric analysis of composition of bio-crude and aqueous phase from hydrothermal liquefaction of thermally and chemically pretreated Miscanthus x giganteus. *Biomass and Bioenergy* **2016**, *95*, 137–145. doi:10.1016/j.biombioe.2016.10.003.

19. Nascimento, I. A., Marques, S. S. I., Cabanelas, I. T. D., Pereira, S. A., Druzian, J. I., de Souza, C. O., Vich, D. V., de Carvalho, G. C., Nascimento, M. A. Screening Microalgae Strains for Biodiesel Production: Lipid Productivity and Estimation of Fuel Quality Based on Fatty Acids Profiles as Selective Criteria. *Bioenergy Res.* **2013**, *6* (1), 1–13. doi:10.1007/s12155-012-9222-2.

20. Islam, M. A., Magnusson, M., Brown, R. J., Ayoko, G. A., Nabi, M. N., Heimann, K. Microalgal species selection for biodiesel production based on fuel properties derived from fatty acid profiles. *Energies* **2013**, *6* (11), 5676–5702. doi:10.3390/en6115676.
